# African Elephants Adjust Speed in Response to Surface-Water Constraint on Foraging during the Dry-Season

**DOI:** 10.1371/journal.pone.0059164

**Published:** 2013-03-15

**Authors:** Simon Chamaillé-Jammes, Godfrey Mtare, Edwin Makuwe, Hervé Fritz

**Affiliations:** 1 Centre d'Ecologie Fonctionnelle et Evolutive, Centre National de la Recherche Scientifique, Unité Mixte de Recherche 5175, Montpellier, France; 2 Scientific Services, Zimbabwe Parks and Wildlife Management Authority, Hwange National Park, Zimbabwe; 3 Laboratoire de Biométrie et Biologie Evolutive, Centre National de la Recherche Scientifique, Unité Mixte de Recherche 5558, Villeurbanne, France; University of Western Ontario, Canada

## Abstract

Most organisms need to acquire various resources to survive and reproduce. Individuals should adjust their behavior to make optimal use of the landscape and limit the costs of trade-offs emerging from the use of these resources. Here we study how African elephants *Loxodonta africana* travel to foraging places between regular visits to waterholes. Elephant herds were tracked using GPS collars during two consecutive dry seasons in Hwange National Park, Zimbabwe. We segmented each individual movement track at each visit to water to define foraging trips, and then used trip-level statistics to build an understanding of movement strategies. Travel speed within these individually-consistent movement bouts was also analyzed to understand if speed was better linked to distance to water or progression in the trip over time. We found that elephants went further from water when drinking less often, which could result from a trade-off between drinking and foraging in less depleted, far from water, places. Speed increased towards the beginning and the end of the trips, and was also greater than observed during the wet season, suggesting that elephants were trying to save time. Numerous short trips traveled at greater speed, particularly when commuting to a different waterhole, was tentatively explained by the inability to drink at specific waterholes due to intra-specific interference. Unexpectedly elephants did not always minimize travel time by drinking at the closest waterhole, but the extra distance traveled remained never more than a few kilometers. Our results show how individuals may adjust movement behavior to deal with resource trade-offs at the landscape scale. We also highlight how behavioral context, here progression in the trip, may be more important than spatial context, here distance to water, in explaining animal movement patterns.

## Introduction

Organisms need to acquire various resources to survive and reproduce. These resources are often patchily-distributed within the landscape, and their degree of spatial correlation may vary. The spatial arrangement of these resources defines the level of landscape complementation [1]. Individuals may adopt complex behavioral strategies to make optimal use of the landscape and limit the costs of trade-offs emerging from the use of several resources (e.g., [2]). Understanding these behavioral strategies is a critical first step to assess the implications of resource distribution across landscapes for animal movements and ultimately population dynamics [3].

Use of surface-water and forage resources by large herbivores in semi-arid landscapes provides a model system to study landscape complementation and behavioral adjustments of individuals facing trade-offs associated with multiple, patchily distributed resources. Indeed, for many species acquisition of these two resources is critical even over short-time scales, as few animals can go without drinking beyond several days, and delayed feeding can only be compensated temporarily by the use of body reserves and rapidly affect physiological processes [4].

Surface-water obviously imposes a large-scale limitation on the distribution of water-dependent species (e.g., [5], [6]), but we surprisingly know little about how wild animals adjust their movement strategies at smaller-scale to meet both forage and water requirements at times when water is scarcely distributed across the landscape [7], [8]. This is a required first step to assess the strength of the trade-off between drinking and foraging and the costs associated to movements from and to water sources [8]. Beyond improving our basic understanding of the ecology of these ecosystems, this may prove critical to design successful water management practices by shedding light on how configuration and density of water sources affect species of interest.

Recent developments of tracking technologies now allow getting accurate description of travel paths of individuals. Thus, we are coming closer to be able to understand the decisions and behavioral adjustments made by animals when facing resource scarcity and/or changes in resource distribution. Often global movement track collected by telemetry are however composed of many movement bouts which represent different behavioral contexts, sometimes linked to environmental conditions (e.g., [9], [10]). We used this conceptual framework to understand how African elephants *Loxodonta africana* adjust behaviorally to the need to both drink and forage in the critical dry season. We thus took a different route than previous studies which looked at how distance to water affects elephants' habitat selection (e.g., [11], [12]).

Here we propose segmenting each individual movement track at each visit to water to define foraging trips between visits to water, and then using trip-level statistics to build an understanding of movement strategies (see also [8] for a somewhat related approach). Movement parameters such as speed within these individually-consistent movement bouts could then be analyzed to understand if movement parameters are better linked to environmental or internal factors.

We applied this framework to the study of dry season elephant movements in a southern-African protected area, Hwange National Park (Zimbabwe). This population is one of the world's largest [13], and its dynamics and distribution within the park is strongly linked to surface-water availability [13], [14]. Elephants aggregate at the few available waterholes during the dry season, which might lead to forage resource depletion around waterholes and reinforce the constraints imposed by the need to forage and drink regularly. Here we addressed 3 questions:

1. how often do elephants drink and do they go further from water when they drink less often?
2. do elephants adjust travel speed to minimize travel time?
3. do elephants drink at the closest waterhole to minimize travel time?

Our results provide a description of dry season waterhole use by elephants in a high density landscape, and strongly suggest that elephants approach the trade-off between foraging and drinking by adjusting movement speed. Our results finally point towards the need to bridge the gap between movement ecology and population dynamics.

## Methods

### Study site

Hwange NP covers c. 15000 km^2^ of semi-arid savanna on the north-western border of Zimbabwe. Mean annual rainfall is c. 600 mm, but rainfall was above-average the two years of the study (781 and 726 mm in 2009 and 2010 respectively; data from Makalolo Camp, Wilderness Safaris). Vegetation is typical of dystrophic Kalahari sands (e.g. *Baikiaea plurijuga*, *Combretum*/*Terminalia* woodlands), but monospecific stands of *Colophospermum mopane* dominates the northern and southern part of the Park where basaltic soils occur [15]. During the wet season numerous rain-fed pools are available to animals and water is very unlikely to constrain space use of water dependent herbivores. Natural surface water is scarce during the dry season, as only few pools remain in the river network, and most natural pans dry up [16]. Artificial waterholes however maintain water availability during the dry season through pumping of underground water (see Figure S1). Dry-season water availability is recorded yearly in September/October by the Wildlife Environment Zimbabwe association, and we used these data here.

The elephant population size in the park has been fluctuating widely around c. 35000 individuals since 1992, after a dramatic increase from c. 13000 individuals in 1986 when annual culling operations were stopped [13]. Elephant dry season density estimated by distance sampling conducted on road transects varied between 0.8 and 2.9 elephants/km^2^ during the study period (HERD program, unpublished data).

### Movement data

The movement data were collected using GPS collars with Inmarsat transmission from the Africa Wildlife Tracking company. Ten collars were deployed in August/September 2009 on elephant cows from 10 different family herds. Due to the limited amount of locations that could be transmitted we used a hierarchical location sampling design to obtain both a long-term, large-scale description of elephant space use and fine-scale daily patterns during selected times of the year: a baseline rate of one location per day was increased to one location per hour during two consecutive weeks in February (wet season) and three consecutive weeks in September/October (dry season). All collars worked for at least two years. When evaluated at a fixed site the GPS relative error was within 30 m ninety-five percent of the time (Chamaillé-Jammes, unpublished data). Data can be accessed on the Movebank website (www.movebank.org) under the dataset named 'African elephant Chamaille-Jammes Hwange NP'.

### Ethics statement

All necessary permits for the study were obtained from the appropriate agency (Zimbabwe Parks and Wildlife Management Authority, permits 23(1)(c) (ii)17/2009; 23(1)(c) (ii) 01/2010; 23(1)(c) (ii)05/2011). Permit applications are reviewed by an ad-hoc committee which considers ethical and animal welfare issues. Immobilization and collaring was carried out specifically for this study by an experienced personnel who is granted authorization to immobilize and collar elephants by the Veterinary Services of Zimbabwe. All operations were conducted under the supervision of Zimbabwe Parks and Wildlife Management Authority ranger. Each collar weighed approximately 13 kg, less than 0.001% of animal body weight.

### Definition of foraging trips

We studied dry season foraging trips using data collected during the 1-hour interval sampling period, and pooled data from 2009 and 2010 to increase sample size as preliminary analyses revealed no significant year effect. Individual movement trajectories were segmented by cutting the trajectory at each visit to a waterhole retaining water, and defining foraging trips as segments of the trajectory that occurred between two consecutive visits at waterholes. We classified each trip as either a looping trip (the elephant drank at the same waterhole at the start and the end of the trip) or a commuting trip (the elephant drank at two different waterholes). Hereafter the waterhole(s) visited at the beginning and at the end of the trips are collectively referred to as start/end waterholes. Due to the 1-hour interval between two GPS locations, we did not always obtain location data when the elephant was exactly at the waterhole drinking. We therefore had to define a threshold distance below which an animal was assumed to have visited the waterhole. If several consecutive locations were at a distance to water shorter than this threshold, the location closest to water was used to end/start consecutive trips. We ran analyses with various thresholds from 0.8 to 1.2 km, on the basis that if for instance elephants stayed 15 min at waterholes accessing water, drinking, bathing, and traveled in straight line but not faster than 3 km/h (over a 1-hour period), a threshold distance of approximately 1 km would allow detecting all visits to water. The various thresholds produced similar results, and analyses with a threshold distance of 1 km are presented here. In 0.8% of the cases the time interval between two successive locations was above 1.5 hours due to GPS failure to acquire a location. In such cases, we calculated at what speed an elephant should have walked to go from the last acquired location to the closest waterhole and back to the current location. If this speed was lower than 3 km/h, we estimated that we could not reject that the focal elephant indeed visited the waterhole, and the current trip was removed from the analyses. The high number of short trips (<12 hours, see *Results*) was unexpected and we visually checked all of them to ensure that they were related to movements from and to water. Only four were uncertain, and these were removed from the analyses. The number of trips retained for analyses was 191.

### Statistical analyses

When classical tests (e.g., chi-square test) were used we simply reported the name of the test and the statistics in the *Results* section. For each trip we calculated the distance traveled, the mean speed, and how far they went from the closest waterhole visited during the trip (hereafter referred to as maximum distance to start/end waterhole). The relationship between these variables and trip type (commuting or looping), trip duration and their interaction were analyzed using linear mixed models. To account for intra-individual correlations elephant identity was used as a random effect on both intercept and slopes. We also studied how speed varied within a trip. We tested if speed was better explained by distance to water, which could be a proxy for forage resource level for instance, or by the progression in the trip (expressed as percent of the total duration of the trip), which could be a proxy for the behavioral context. To do so we built four alternative linear mixed models with distance to water or progression in the trip used as linear or quadratic predictors. Trip type and trip duration were entered as additional explanatory variables in these models, and elephant identity was used as a random effect on intercept (some models did not converge when using random slopes; for those converging results were similar between models having random intercept and slopes or random intercept only). We compared these models using the Akaike Information Criterion (AIC) and reported the parameter estimates from the model with the lowest AIC.

For all models described above we obtained 95% confidence intervals of the parameter estimates using a parametric bootstrap based on 10000 random samples [17]. Parameters for which 95% confidence intervals did not include zero were considered significant. Models were fitted using the *lme4* package [18] in R [19].

## Results

**Figure 1:**
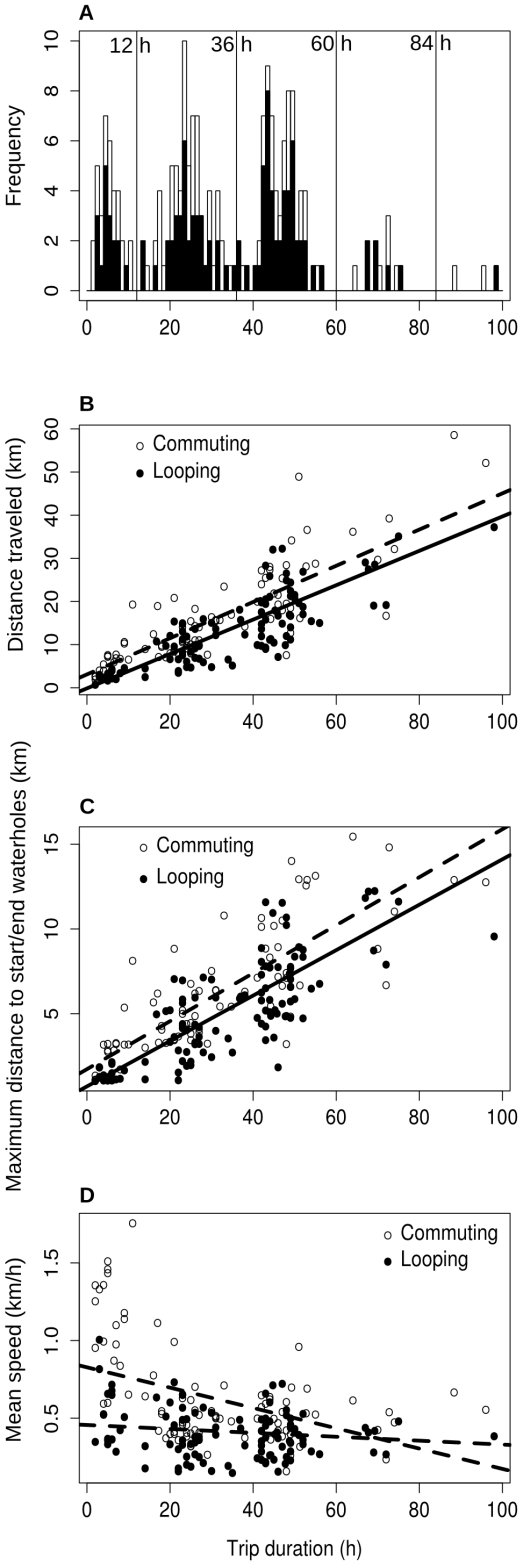
Characteristics of trips conducted between visits to waterholes. (A) Frequency of trip durations; (B) Relationship between distance traveled and trip duration; (C) Relationship between the maximum distance to waterholes visited at the start/end of the trip and trip duration; (D) Relationship between mean travel speed and trip duration. Note that in the wet season when elephants are not constrained by water availability mean speed is 0.33±0.52 s.d. km/h. All panels show differences between commuting trips (when different waterholes are visited at the beginning and at the end of the trip; white-filled symbols, dotted lines) and looping trips (when the same waterhole is visited at the beginning and at the end of the trip; black-filled symbols, solid lines). Note that the difference between commuting and looping trips in panel (C) is not significant (see Table 1).

The duration of trip between two visits at waterholes was clearly multi-modal with peaks around 6, 24, 48 (with two sub-peaks at 44 and 48 h), and 72 h (Fig. 1A). The proportions of trips lasting 12 hours or less, between 12 and 36 hours, between 36 and 60 hours, between 60 and 84 hours, and over 84 hours were 16.7%, 38.2%, 37.7%, 5.8% and 1.6% respectively. The distribution of trip durations did not differ between commuting and looping trips (Chi-square test, P = 0.220).

Increasing trip duration was associated with greater distance traveled (Fig. 1B, Table 1A) and greater maximum distance to start/end waterhole reached (Fig. 1C, Table 1B). For the same duration distance traveled was on average greater in commuting trips because they were (on average) traveled at higher speed than looping trips (Fig. 1D, Table 1C). The differences in distance traveled remained small however because the difference in speed was most important – up to more than twice – only in short trips (Fig. 1D).

**Table 1:** Factors affecting the characteristics of trips conducted between visits to waterholes.

	Estimate [95% C.I.]
**A. Distance traveled (km)**	
Looping	−0.271 [−2.259/1.771]
Commuting	**3.247 [0.583/5.900]**
Trip duration (h)	**0.402 [0.339/0.463]**
Commuting * Trip duration	0.021 [−0.050/0.091]
**B. Maximum distance to start/end waterhole (km)**	
Looping	0.714 [−0.023/1.456]
Commuting	1.019 [−0.088/2.127]
Trip duration (h)	**0.134 [0.106/0.162]**
Commuting * Trip duration	0.008 [−0.029/0.045]
**C. Speed (km/h)**	
Looping	**0.456 [0.346/0.569]**
Commuting	**0.371 [0.199/0.541]**
Trip duration (h)	−0.001 [−0.003/0.001]
Commuting * Trip duration	−**0.005 [**−**0.009/**−**0.002]**

Distance traveled (A), maximum distance to start/end waterhole (B) and mean speed (C) were regressed against explanatory variables in linear mixed models with elephant identity as a random effect on intercept and slopes. The waterhole at the beginning of the trip is either different (commuting trip) or the same (looping trip) than the waterhole at the end of the trip. Estimates of the reference intercept (looping trips) and of deviations associated to other levels of explanatory variables are presented, with 95% confidence intervals obtained by parametric bootstrap with 10000 samples. Estimates for which the 95% confidence interval do not include zero are in bold.

**Figure 2:**
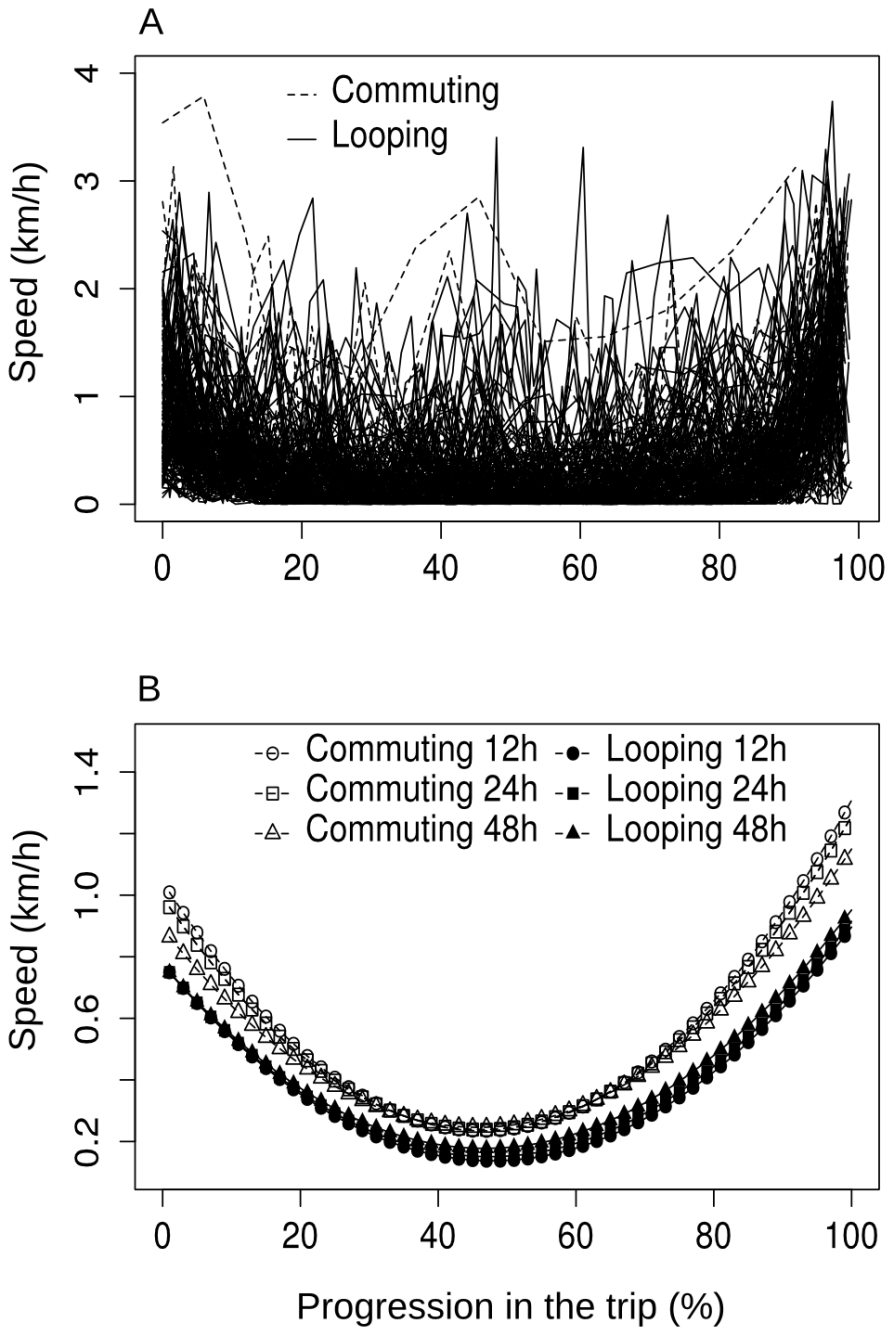
Relationship between speed and progression in the trip. (A) Speed of elephants within trips, in relation to the progression in the trip (expressed as percent of the total duration of the trip). Each line represents data for a single trip. Trips are defined as the movement between visits to waterholes, and elephants are therefore closer to water at the beginning and at the end of the trip. The waterhole at the beginning of the trip is either different (commuting trip) or the same (looping trip) than the waterhole at the end of the trip. A statistical model revealed that speed was best related to progression in the trip, trip type (commuting vs. looping) and some interactions with trip duration (see Table 2). Panel (B) shows the model predictions. Note that in the wet season when elephants are not constrained by water availability mean speed is 0.33±0.52 s.d. km/h.

Remarkably, speed was better predicted by the progression in the trip than by distance to water. A model incorporating a quadratic relationship between progression in the trip and speed fitted the data best (model with linear effect of distance to water, AIC = 8709; model with quadratic effect of distance to water, AIC = 8865; model with linear effect of progression in the trip, AIC = 8914; model with quadratic effect of progression in the trip, AIC = 7714). Table 2 presents the estimates from this model, and data and model predictions are shown in Fig. 2A and 2B respectively. On average speed was thus higher at the beginning and particularly at the end of trips, for both commuting and looping trips (Fig. 2). Speed was also higher on average in the dry season than in the wet season (t-test, P<0.001), during which mean speed was 0.33±0.52 s.d. km/h, with 0.8, 1 and 1.2 km/h achieved only 9.7, 7.7 and 6% of the times respectively.

**Table 2:** Factors affecting elephant speed between visits to waterholes.

	Estimate [95% C.I.]
Looping	**0.777 [0.638/0.916]**
Commuting	**0.318 [0.147/0.489]**
Trip duration (h)	−4.446e-5 [−2.624e-3/2.490e-3]
Progression	−**0.027 [**−**0.033/**−**0.021]**
Progression^2^	**2.810e-4 [2.230e-4/3.385e-4]**
Progression * Commuting	−**0.010 [**−**0.018/**−**0.002]**
Progression * Trip duration	3.250e-5 [−8.761-5/1.535e-4]
Progression^2^ * Commuting	**1.161e-4 [3.391e-5/1.972e-4]**
Progression^2^ * Trip duration	−1.662e-7 [−1.387e-6/1.039e-6]
Trip duration * Commuting	−**0.004 [**−**0.008/**−**5.797e-4]**
Progression * Trip duration * Commuting	1.516e-4 [−1.697e-5/3.199e-4]
Progression^2^ * Trip duration * Commuting	−**1.694e-6 [**−**3.376e-6/**−**1.100e-8]**

Speed was regressed against explanatory variables in linear mixed models with elephant identity as a random effect on intercept. The 'Progression' variable was included as a quadratic predictor (i.e. the value and the square of the value ('Prediction^2^') was included in the model). The waterhole at the beginning of the trip is either different (commuting trip) or the same (looping trip) than the waterhole at the end of the trip. Progression in the trip was expressed as percent of the total trip duration. Estimates for the reference intercept (looping trips) and for the deviations associated to other levels of explanatory variables are presented, with 95% confidence intervals obtained by parametric bootstrap with 10000 samples. Estimates for which the 95% confidence interval do not include zero are in bold.

Elephants did not always drink at the closest waterhole, as the waterhole they went to drink to at the end of a trip was at a greater distance (on average 2.03±2.00 s.d. km) than the closest waterhole in 38.9% of the trips.

## Discussion

We have investigated here how elephant herds use water sources during the dry season when water exerts the strongest constraint on movement across the landscape. A notable result of our study is that elephants increased their speed when leaving from or arriving at waterholes. Taken alone this might only indicate that both foraging and drinking places are well identified and that elephants try to commute rapidly between them. Apart from during the middle of trips, speed was noticeably greater than in the wet season however. This was particularly true for commuting trips, for which *average* speed during the first and last 20% of the trips – as presented by the model predictions in Fig. 2B – was rarely achieved in the wet season even over any 1-hour interval. Higher speed is likely to be associated with higher costs, such as increased heat stress, particularly at this time of the year when hot temperature are experienced, or increased risk of loosening of the female-juvenile spatial bond [20]. This suggests that elephants tried to minimize time lost commuting between food and water. How elephants used the time saved this way is unknown, but could likely be spent on additional travel time allowing reaching areas that are further from water, while maintaining reasonable duration between two drinking events. Indeed, within the dry season longer periods spent without drinking (up to 3 or 4 days, herds rarely drink less often in the wild) are associated with trips reaching greater distance to water. Thus, elephants clearly face a trade-off between foraging far from water and drinking every 24 h or 48 h, as they usually do when remaining close to water. We do not yet know if foraging far from water allows elephants reaching better foraging places, but we suspect so. Despite a great spatial heterogeneity in woody cover, there is a significant increase in woody cover with distance to water in Hwange NP [21]. This spatial trend in food availability (elephants are mostly browsers in the dry season) linked to long-term habitat change may also be greatly reinforced by depletion of the forage resources close to water as the dry season progresses, forcing elephants to move further from water. The assumption of a depletion occurring in the vicinity of water is common in other elephant studies [22], but to the best of our knowledge remains to be demonstrated with behavioral observations.

Elephants could save travel time by drinking at waterholes closest to foraging places, but this was not the case in almost 40% of the trips. Note however that the traveling distance elephants would have saved by drinking to the closest waterhole - rather than to the one they actually drank from - was rarely more than a few kilometers. Such short distances relative to elephant mobility, in conjunction with their intense social communication, make it unlikely that this use of waterholes is due to a lack of spatial knowledge. It is more likely that various factors – still unknown, but which could for instance be related to neighboring habitats or water quality [23] – cause some waterholes to be selected or avoided. Determining these factors will be critical to be able to predict redistribution of elephants facing management or environmental (e.g. climate) change which will cause waterhole closure or dry-up.

An unexpected result of our study was the relatively large number of short trips (<12 h), which is unreported in the literature. Although we cannot reject the hypothesis that these trips were indeed short foraging trips, we suspect most of them occurred when local conditions were considered inappropriate for drinking at the first waterhole visited, and elephants went to drink at another waterhole. For instance, field observations show that during the dry season it is not uncommon to see herds waiting to access clean water at the through because of dominant males or herds, or simply because of a crowding effect preventing access to water. In such case, elephants may decide to either postpone drinking and come back later or go to another waterhole located close-by. The observation that short commuting trips were conducted at higher speed than any other trips support the idea that elephants are trying to make up for the time spent traveling. If true – field observations of tracked elephants are now required – this pattern of many short trips may be a sign of negative density dependent effects in a context of limited water availability, and would be a proximate mechanism to explain why elephants started using less crowded waterholes after culling stopped and population size increased [14].

Several aspects of our research could be improved upon in the future: first, higher sampling frequency would increase the certainty of waterhole visits, although similarity of results obtained with various distance threshold and visual analyses of the trips helped us build confidence in our results. The limitations imposed by collar battery life have now been removed in new-generation collars. Second, field data of elephant activity should be gathered simultaneously to the acquisition of tracking data. This would for instance ascertain the local elephant abundance at waterholes at the time of visits, or the resource availability at feeding sites at various distance to water. Only with the simultaneous observation of local conditions and movement trajectory before/after observations will one be able to fully grasp with confidence the environmental determinants of elephant movements.

Overall, our study provides evidence that time represents an important currency used by elephants to address the trade-off between foraging and drinking. The generalization of this statement to other species has to be investigated. Buffaloes or zebras are often found close to water when water is scarce [7], [8]. On the opposite, sable antelopes avoid concentration of other water-dependent grazers by foraging far from water and thus incur larger time costs of traveling to/from water [8]. Few other species have been studied in that regard. To determine the costs of drinking a conceptual framework including species physiology, species interactions and distribution of suitable habitat patches within the landscape can be envisioned, but data are lacking to validate this framework. Current tracking studies will shed light on this, and should ultimately quantify the fitness costs of travels to water. Researchers should now try to bridge the gap between movement ecology and population demography. We have also revealed how behavioral context, represented here by progression in the trip, may be a better predictor of movement parameter than spatial context or environmental factors, here distance to water. This strongly calls for a deeper integration of behavioral context in the analysis of animal movement.

## Supporting Information

Figure S1 **Map of the study area.** Black dots represents dry-season waterholes. In the wet season water is virtually available everywhere in rain-fed pools. The solid line shows the boundary of Hwange National Park.
(TIF)
