# Supplementary figures and images for: African Elephants Adjust Speed in Response to Surface-Water Constraint on Foraging during the Dry-Season
Source: PLoS One. 2013 Mar 15;8(3):e59164. doi: 10.1371/journal.pone.0059164 (PMC3598744; doi:10.1371/journal.pone.0059164)

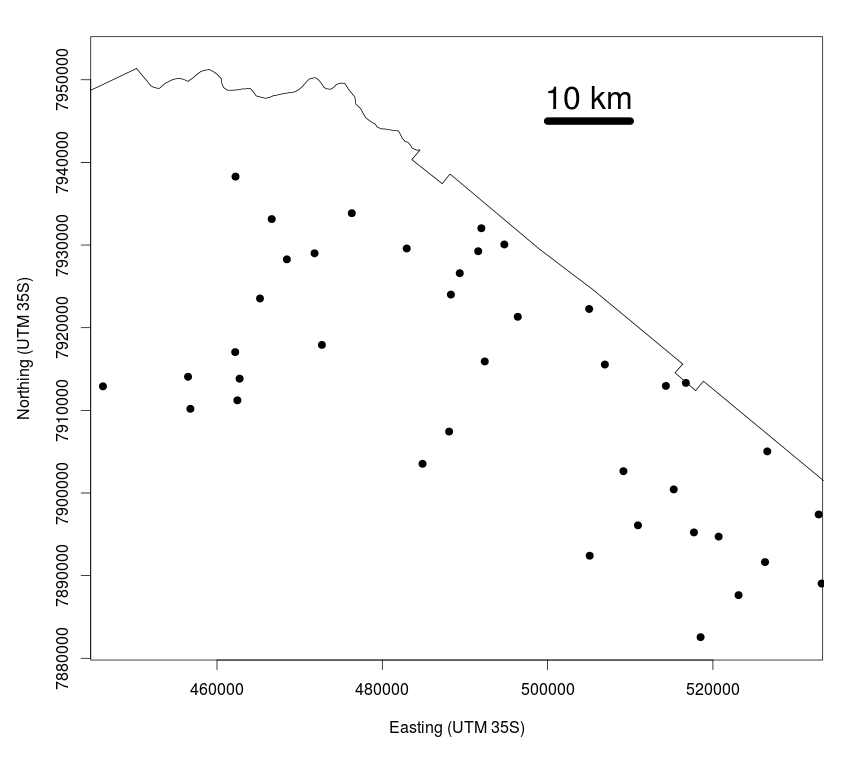

Supplement: Figure S1 — Map of the study area. Black dots represents dry-season waterholes. In the wet season water is virtually available everywhere in rain-fed pools. The solid line shows the boundary of Hwange National Park. (TIF) [file pone.0059164.s001.tif]
